# Points

**Published:** 1898-10

**Authors:** 


### POINTS.

A yearling Shropshire ram, prize-winner at the royal show, recently sold for $1650.

A gray draught-gelding, representing a cross between the Shire and Percheron, and weighing 2650 pounds, was recently sold in the Chicago stockyards for export to Liverpool. He brought $350.

Birmingham, Alabama, through its Central Abattoir and meat-inspection has increased the consumption of meat and not advanced the cost to the consumer.

Officials of the Department of Agriculture are sanguine of the success of the proposed serum-treatment measures for dealing with hog-cholera. The work and its results will be keenly watched by those whose losses have been so heavy from this scourge.

A Mexican burro at the Cheyenne Cañon and Seven Falls, Colorado Springs, Colorado, is said to have attained the age of fifty-five years; it was toothless, and met death by a trolley car.

Colorado Springs, Colorado, finds employment for some six veterinarians. The work of City Veterinarian, formerly done by the late Dr. D. P. Frame, is now divided among several of the city's veterinarians.

Denver, Colorado finds employment for some sixteen veterinarians. The enhancing value of horses is adding to their increased employment and prosperity.

The Paris Municipal Council is now considering the advisability of building a special slaughter-house for horses, on account of the continual increase in the consumption of horse-flesh. The first horse-butcher shop was opened in 1866, and in the following year 2152 horses were consumed. Last year 14,840 horses, 275 donkeys, and 40 mules, making a total of 15,155 animals, weighing 2,743,000 kilos, were eaten tby the Parisians.

After several Pittsburg and Allegheny veterinarians had been buncoed by alluring calls into the country and given up some of their hard-earned dollars to the steerer, they combined, laid a trap, caught the offender and jailed him for ninety days. A search of the prisoner brought forth a number of prominent veterinarians' visiting-cards in many cities, showing he was feeding on and seeking fresh pastures frequently.

Large numbers of cattle in Nebraska, Colorado, South Dakota, Texas, and Kansas have been vaccinated for black-leg this year, using the Pasteur vaccine for this purpose. Last year the losses in the districts where it was used was insignificant, the highest being 1 per cent. in a herd of 2400 cattle.

Letters patent have been granted to one of the makers of antitoxins; many of the others whose preparations are in the market are preparing to defend the same in open court. Surely our Government, through its Patent Office, does not want to place such a premium or restriction upon these remedies as to limit their use in the relief of suffering and the saving of lives.

The Illinois Board of Livestock Commissioners are asked to bear the cost of inspection of actinomycotic cattle at the Chicago stockyards, which is now borne by the commission merchants.

